# Climate Change and Simulation of Cardiovascular Disease Mortality: A Case Study of Mashhad, Iran

**Published:** 2017-03

**Authors:** Mohammad BAAGHIDEH, Fatemeh MAYVANEH

**Affiliations:** Dept. of Physical Geography, Faculty of Geography and Environmental Sciences, Hakim Sabzevari University, Sabzevar, Iran

**Keywords:** Climate change, Cardiovascular, General circulation model, Maximum temperature

## Abstract

**Background:**

Weather and climate play a significant role in human health. We are accustomed to affects the weather conditions. By increasing or decreasing the environment temperature or change of seasons, some diseases become prevalent or remove. This study investigated the role of temperature in cardiovascular disease mortality of city of Mashhad in the current decade and its simulation in the future decades under conditions of climate change.

**Methods:**

Cardiovascular disease mortality data and the daily temperatures data were used during (2004–2013) period. First, the correlation between cardiovascular disease mortality and maximum and minimum temperatures were calculated then by using General Circulation Model, Emissions Scenarios, and temperature data were extracted for the next five decades and finally, mortality was simulated.

**Results:**

There is a strong positive association between maximum temperature and mortality (r= 0.83, *P*-value<0.01), also observed a negative and weak but significant association between minimum temperatures and mortality. The results obtained from simulation show increased temperature in the next decades in Mashhad and a 1 °C increase in maximum temperature is associated with a 4.27% (95%CI: 0.91, 7.00) increase in Cardiovascular disease mortality.

**Conclusion:**

By increasing temperature and the number of hot days the cardiovascular disease mortality increases and these increases will be intensified in the future decades. Therefore, necessary preventive measures are required to mitigate temperature effects with greater attention to vulnerable group.

## Introduction

The human body is affected by the thermal environment, influenced by many different factors air temperature, radiant temperature, humidity and air movement are the four basic environmental variables that affect human response to thermal environments (1–3). The relationship between high temperatures and the increase in mortality and disease rates has been described in detail for all over the world (4, 5). Usually days with high and low temperatures have relationships with mortality rate (6–9). The relationship between temperature and mortality had been extensively studied in Europe (10), United States (11–14) Australia (15–21), Korea (22) and Iran (24). For example, the change in all natural mortality associated with 1 °C increase in maximum temperature above the city-specific threshold was 3.12% (95% credibility interval = 0.60% to 5.72%) in the Mediterranean region and 1.84% (0.06% to 3.64%) in the north-continental region. Evaluating the association between ambient air temperature and specific health outcomes can help in identifying vulnerable populations and formulating preventive actions (25).

Cardiovascular diseases are considered as the first causes of morality in the world and the morality caused by these diseases are much more than any other causes (26). Several studies have examined the effects of air temperature on overall cardiovascular mortality (10, 27). The relationship between temperature and morality caused by cardiovascular diseases in forms of v, u, and j. This issue indicates that the risk of mortality of cardiovascular diseases increases for days with hot and cold temperatures (28).

According to the UN’s Intergovernmental Panel on Climate Change (IPCC) Fourth Assessment Report, climate change is likely to affect human health directly through changes in temperature and precipitation and indirectly through changes in the ranges of disease vectors (e.g., mosquitoes) and other channels (29). Climate change is potentially the biggest global health threat in the 21st century (17, 30). Future climate change will increase the frequency, intensity, and duration of heat waves (31). Not only has the global average temperature increased, but the frequency and intensity of extreme temperatures (e.g., heat waves and cold spells) have, also, the projected changes in surface air temperature in West Asia range between 1.26 and 6.3 °C over the period 2010–2099 (with respect to the baseline period (1961–1990) for the B1 and A1F1 scenarios (32).

The projected temperature rise, along with higher frequency and intensity of heat waves, is expected to increase heat-related premature mortality and illnesses (33, 34). A growing number of studies have projected future heat-related mortality due to climate change in recent years (35–37).

The heat-related mortality were estimated in the UK in the 2020s, 2050s, and 2080s (38). The present-day relationship between daily mean temperature and mortality rates will apply in the future, and have applied an ensemble of climate models working from the Special Report on Emissions Scenarios (SRES) A1B emissions scenario. Using the same method (39) investigated future heat-related mortality impacts in six cities (Boston, Budapest, Dallas, Lisbon, London and Sydney), they showed that higher mortality is attributed to increases in the mean and variability of temperature with climate change rather than with the change in mean temperature alone. Besides, Heatwave considered under three different climate change scenarios for 2081–2100 and in the absence of adaptation (40). Their results showed city of Chicago, Illinois could experience between 166 and 2217 excess deaths per year attributable to heat waves, based on estimates from 7 global climate models. Regional adaptation planning is unfortunately often limited by the lack of quantitative information on potential future health responses. Studies in this field are limited in Iran and a lot of them have been conducted outside Iran. In addition, the relationship between climatic parameters and the mortality rate has been considered, while the issue of the climate change and its effects on the mortality rate has received less attention. Regarding the issue, that Mashhad city is one of the most populated cities in Iran and has very different climatic conditions in different seasons of a year, and like other cities in the word, it has been influenced by conditions of climate changes in the future.

The aim of the present research was to assess and report on future cardiovascular disease mortality in Mashhad, potentially leading to improved understanding of weather and climate vulnerability in the health sector, and more informed risk management and adaptation decisions. Generally, there is an urgent need to evaluate the relationship between climate change and human health, to better identify vulnerable populations and take preventive measures. In this paper, we investigated the association between temperature and cardiovascular disease mortality than mortality for the next five decades is simulated.

## Materials and Methods

### Study area

**Fig. 1:**
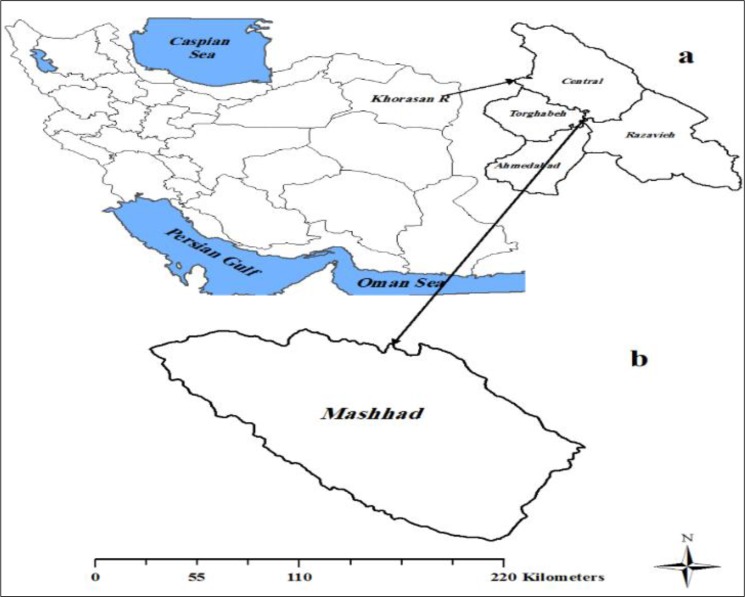
The geographical location of the study area in Iran (a) Shows the location of Khorasan Razavi Province in Iran, (b) is the location of Mashhad in Khorasan Razavi Province

Mashhad is the populous city (after Tehran) in Iran (3069941) and is the capital of Khorasan Razavi Province, located in the north-east of the country (Fig. 1). The city is located at 36.20° North latitude and 59.35° East longitude, in the valley of the Kashaf River between the two mountain ranges of Binalood and Hezar-masjed. Mashhad features a steppe climate (Köppen BSk) with hot summers and cool winters. Summers are warm with average temperatures above 30 °C. However, real extremes in temperatures such as temperatures above 40 °C.

In the present study, mortality data of cardiovascular diseases from database of Mashhad municipality were used according to the International Classification of Diseases and Related Health Problems 10^th^ Revision (ICD-10) including codes 100–199 (41), for (2004–2013) period. Daily meteorological variables of maximum and minimum temperature (°C), precipitation (mm) and solar radiation (MJm^−2^day^−1^) for Mashhad were obtained from the Iran Meteorological Organization, in (1986–2005) period.

### Statistical analysis

Then, by using Pearson correlation model, the relationship between temperature (minimum and maximum) and cardiovascular mortality was studied and the Simple Linear Regression Model was used to simulation of mortality in the future decades.

(y=0.2166x+0.8814)

Where x is the independent variable (max temperature) and y is the dependent variable (mortality). In the following, by using LARS-WG model, data of the General Circulation Models (GCMs) (in this study, HadCM3), under emission scenarios (in this study, A2) confirmed by IPCC were downscaled and the data of max and min temperatures were simulated for the future decades (2021–2030, 2046–2055, 2056–2065, 2080–2089, 2090–2099). Finally, by using the Linear Regression Model cardiovascular mortality were simulated.

### Atmospheric General Circulation Models

Atmospheric GCMs are mathematical models based on numerically discretized versions of differential equations that describe the atmospheric physics and dynamics, utilized to simulate the global atmospheric circulation.

### Description of LARS-WGM model

LARS-WG is a stochastic weather generator and is used for simulating weather data at a single site under both current and future conditions (42–46). LARS-WG uses observed daily weather data for a given site to compute a set of parameters for probability distributions of weather variables as well as correlations between them, used to generate synthetic weather time series of arbitrary length by randomly selecting values from the appropriate distributions. For each climatic variable *v* value of a climatic variable v_i_ corresponding to the probability p_i_ is calculated as:

$$v_{i}=\text{min}\{v:p(\nu_{\mathrm{obs}}\leq \nu )\geq \rho_{i}\}i=0,...,n$$

Where *p*(*v_obs_* ≤ ν) denotes probability based on observed data {*v_obs_*}. For each climatic variable, two values, *p*_0_ and *p_n_*, are fixed as *p*_0_ = 0 and *p_n_* = 1, with corresponding values of *v*_0_ = min{*v_obs_*} ((47, 48)). The data utilized in the form of daily time series for suitable climate variables are precipitation (mm), maximum and minimum temperature (°C), and solar radiation (MJm^−2^day^−1^) (45, 49) of climatic variables and correlations between them derived from observed daily weather data at a given site for a long-term period (50, 51). To evaluate the produced data by the model and the observed data, statistical parameters such as coefficient of determination (R^2^) and Root Mean Square Error (RMSE) were used and the results are shown in Table 1.

$$\mathrm{RMSE}=\sqrt{\frac{\sum_{i=1}^{n}{\left(X_{\mathrm{obs},i}-X_{\mathrm{model},i}\right)}^{2}}{n}}$$

$$R^{2}=\left[\sum_{i=1}^{n}\left(X_{i}-\bar{X}\right)\left(Y_{i}-\bar{Y}\right)\right]/\sum_{i=1}^{n}{\left(X_{i}-\bar{X}\right)}^{2}\sum_{i=1}^{n}\left(Y_{i}-\bar{Y}\right)$$

**Table 1:** Statistical parameters (R2 and RMSE) for Model Validation and Calibration

Statistic parameter	Max Temperature	Min Temperature
R^2^	0.9976	0.9966
RMSE	0.2710	0.4370

After calibration of the model and confirmation of its ability in simulation of climatic parameters, of the basic period (1986–2005), with regard to the behavior of the climate in the basic period, and statistical downscale of data of a General Circulation Model, future climatic parameters were simulated.

### Hadley GCM 3 model

In this study, the output from the Hadley GCM 3 model (HadCM3) was utilized. It uses a 360 d per year and has a spatial grid with dimensions 2.5° latitude × 3.75° longitude. This GCM contains a complex model of land surface processes. It is considered the most mature and popular of the GCMs. This GCM is unique, in which it does not require flux adjustments to produce a realistic scenario (52, 53).

### Emissions scenarios

Emissions scenarios describe future releases into the atmosphere of greenhouse gasses, aerosols, and other pollutants and, along with information on land use and land cover, provide inputs to climate models (54, 55). They are based on assumptions about driving forces such as patterns of economic and population growth, technology development, and other factors.

### Scenario, A2

The A2 storyline and scenario family describes a very heterogeneous world. The underlying theme is self-reliance and preservation of local identities. Fertility patterns across regions converge very slowly, which results in continuously increasing population. Economic development is primarily regionally oriented and per capita economic growth and technological change more fragmented and slower than other storylines (57, 58).

## Results

### Cardiovascular disease mortality

**Fig. 2:**
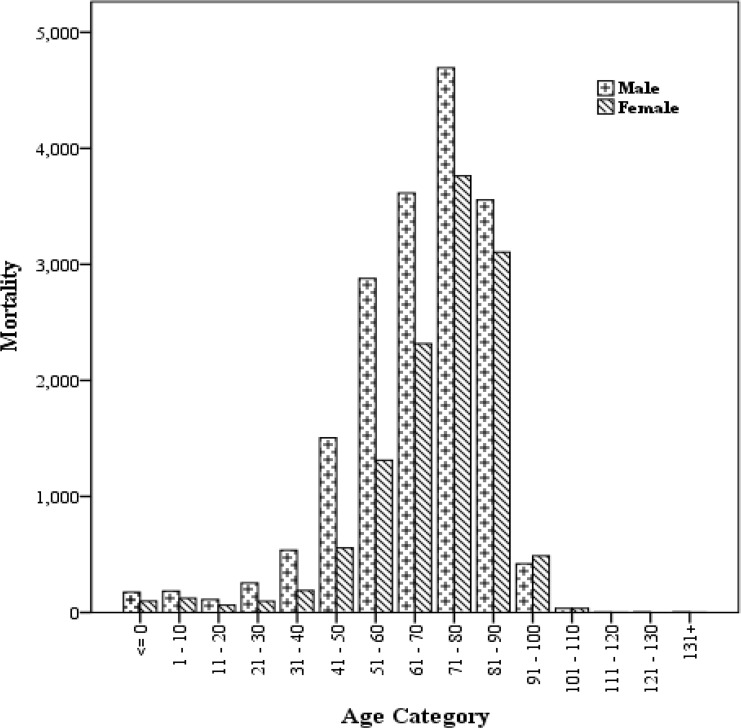
Cardiovascular diseases mortality between male and female for different age groups in Mashhad during the baseline period (2004–2013)

According to demographic characteristics of study population, total number of cardiovascular disease mortalities in Mashhad was 30121people in 2004–2013 periods that most of them (8585 people) were in the (72–81) age group (Table 2). In the present study, chi-squared test was used to evaluate the cardiovascular disease mortality rate among women and men. The results showed, maximum mortality has happened among women and men in 72–81 age group (Fig. 2).

**Table 2:** Socio-demographic features of the study population (n = 30121)

**Variable**	**Total (n=30121)**	**Men (n=17972)**	**Women (n=12149)**
**Age groups (yr)**<=0	273	174	99
1–10	307	184	123
11–20	174	111	63
21–30	351	254	97
31–40	726	536	190
41–50	2061	1505	556
51–60	4190	2878	1312
61–70	6650	4039	2611
71–80	8585	4698	3887
81–90	6053	3235	2818
91–100	679	318	361
101–110	58	28	30
111–120	4	3	1
121–130	1	9	1
**Temperature (°C)**	Min	−14.65	_
	Max	33.25	
	Mean	23.95	
	SD	9.57	
	VAR	91.66	

SD: Standard Deviation

VAR: Variance

### Relationship between temperature and cardiovascular mortality

In order to evaluate the relationship between max and min temperatures and cardiovascular disease mortality, Pearson correlation model was used. There is a relationship between cardiovascular mortality and max temperatures which are positive and strong for temperatures more than 26 °C (r=0.83, *P*<0.01) such that, 1 °C increase in maximum temperature is associated with a 4.27% (95%CI: 0.91, 7.00) increase in cardiovascular disease mortality. In addition, there observed a negative and weak (r=0.47) but significant (*P*<0.01) association between minimum temperatures and mortality.

### Simulation of temperature and mortality in the future decades

**Fig. 3:**
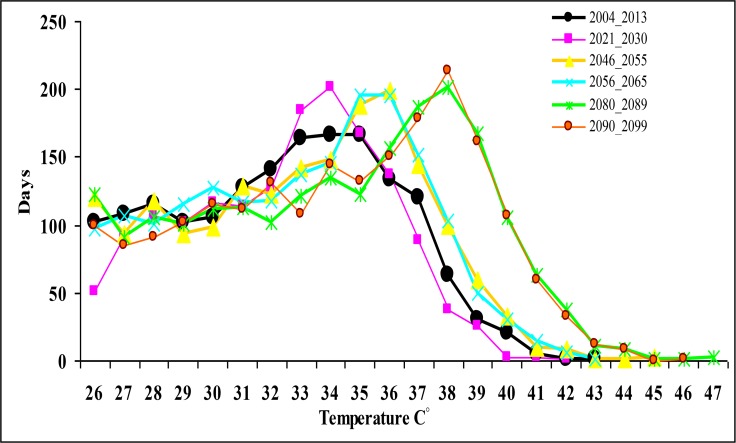
Number of days with max temperatures higher than 26 °C during future decades compared to the baseline period

According to Table 1, the LARS-WG model has a reasonable capability of simulating the minimum and maximum temperatures. In the present study temperatures, more than 26 °C (threshold temperature) were simulated for the future decades by LARS-WG model. The comparison between the observed decade and the future decades shows the frequency of days with max temperatures higher than 26 °C will be a significant increase for the next five decades (especially in 2089–2080 and 2099–2090) (Fig. 3).

After simulation of max temperature in the future decades, by using Linear Regression Model (*y* = 0.2166*x* + 0.8814) that was obtained in the observed decade, mortality was simulated under emission scenario A2.

**Fig. 4:**
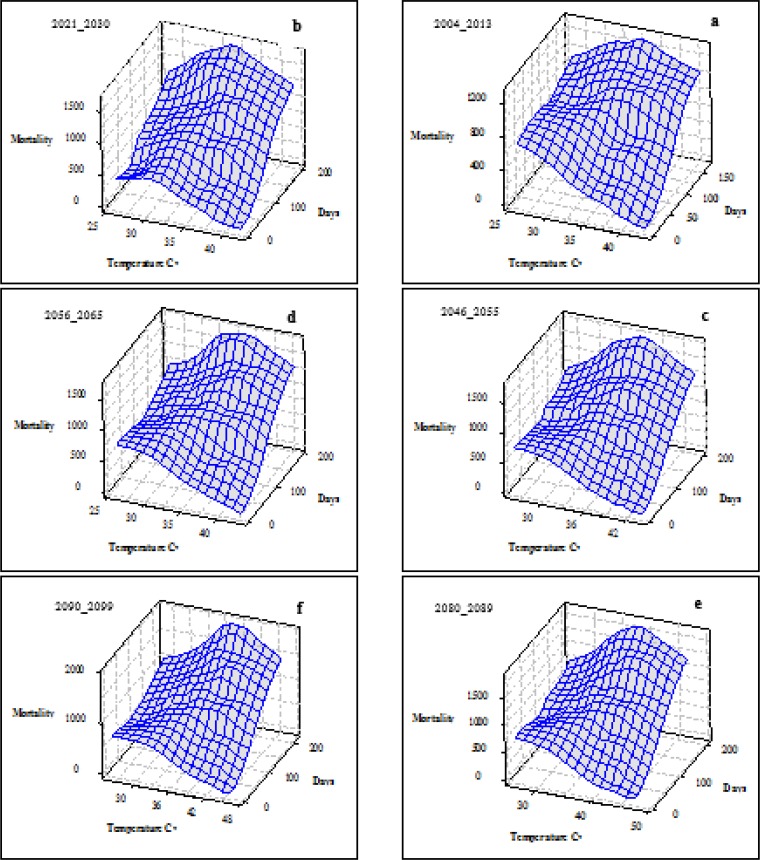
Mortality and frequency of hot days (above 26 °C), observed decade (a) and future decades (b–f)

By increasing the temperature in the future, decade’s mortality will also increase. In Fig. 4, temperature, frequency (days) and number of mortality are shown for the observed period (a) and the future decades (b to f). In the observed period, max temperature was 43 °C, maximum frequency was recorded in 34 °C, and maximum mortality is seen at this temperature. For the future decades, in addition to the temperature increase the frequency of temperatures more than 26 °C (threshold temperature) has also increased which made the increase of mortality. Maximum increases will be seen for 2080–2090 such that the maximum estimation is 47 °C (e) which leads to the highest rate of mortality.

## Discussion

Cardiovascular disease mortality in women is much smaller than men of less than 71 yr of age in study area, while for most of the developed countries this age is 45 yr (59–61). It seems to be the cause of this difference being lower standard of living and quality of life in the developing countries.

Among men, CVD mortality increases rapidly after age of 30 yr and reaches to the highest mortality at the age of 71. In the age groups above 71, mortality among women increased significantly compared to men. Therefore, there are sex-related differences in cardiovascular disease mortality. This difference has been largely attributed to circulating estrogens present in women of fertile age. This explanation can be supported by epidemiological data that early menopause occurring spontaneously or as a result of bilateral oophorectomy is associated with 4–7 fold elevation in the risk of CVD (63). However, other explanations for high male CVD mortality at young ages may well exist. Various CVD risk factors, such as smoking, heavy drinking or poor dietary habits accumulate in young men, perhaps to the degree that CVD ensues (63).

In this paper, we investigated the association between air temperature (maximum and minimum) and cardiovascular disease mortality in Mashhad, Iran, during the years 2004–2013. The influence of the temperature (both minimum and maximum) on mortality was confirmed. This relationship in many regions of the world is proven (64–67). In this study observed a strong and positive association between cardiovascular mortality and maximum temperature. While this relationship, was negative and weak but significant for minimum temperature. In this way, maximum temperature was used for detailed review. The most researchers conducted in relation with the effect of thermal parameters on cardiovascular disease mortality have significantly emphasized this relationship. This is while this relationship sometimes has been in reverse direction regarding the rate of mortality due to other diseases such as respiratory diseases. For example, with the increase in the temperature, the rate of mortality reduces, and with the decrease in the temperature, it rises (1).

According to Fig. 3 and 4, the number of days with temperatures above the threshold in the coming decades compared to the observed decade increases. In addition, in the observed period, the highest frequency is related to 34 °C. In the 2021–2030 decade, the highest frequency is related to the same temperature, but in the 2046–2055 and 2056–2065 periods also known as the “middle-future” periods, the highest temperature is related to 36 °C (as 2 °C increase). The prediction of the highest frequency is based on the 2080–2089 and 2090–2099 decades as future periods, the temperature will be 38 °C. Furthermore, the highest estimated temperature in the future decades compared to the observed period indicates 5 °C increase, which can be an important issue from the perspective of human beings’ health.

High temperatures can increase the occurrence of heart attacks and strokes in susceptible patients because of increased blood viscosity (68). Heat can induce events such as heart failure or stroke. Proposed mechanisms between heat and cardiovascular mortality include increased surface blood circulation and sweating. This leads to increased cardiac workload, dehydration and salt depletion, haemoconcentration, elevated blood viscosity, and the risk of thrombosis (69) Moreover, heat stress was suggested to induce the release of interleukins modulating local and systemic acute inflammatory responses (69). These inflammatory responses can result in heart failure by increasing damage to heart tissue and inflammation (70). Significant increases in extreme heat are projected to continue in coming decades, consistent with observed global trends in past decades (71). The A2 scenario was selected to cover a wide range of temperature rise. This scenario family describes a very heterogeneous world. The use of other scenarios can provide the possibility of comparing and estimating diverse more conditions future. The underlying theme is self-reliance and preservation of local identities. Fertility patterns across regions converge very slowly, which results in continuously increasing global population. Economic development is primarily regionally oriented and per capita, economic growth and technological change are more fragmented and slower than in other storylines (59). In the next decades, temperature increases and the frequency of hot days will also increase. These results suggest that more CVD mortality can be expected in the future.

### Limitations of study

The most important limitation of this study was the lack of long-term mortality data that led the observation period was limited to only one decade. Therefore, we could not study the effects of climate change on mortality in the past decades (For example, the last half century) very well.

## Conclusion

There is a strong positive association between maximum temperature and cardiovascular disease mortality. Besides, there observed a negative and weak but significant association between minimum temperatures and mortality. The results obtained from simulation show increased temperature in the next decades in Mashhad. In general, increasing temperature and the number of hot days the Cardiovascular will intensify mortality increases and these increases in the future decades. Therefore, necessary preventive measures are required to mitigate temperature effects with greater attention to vulnerable group.

## Ethical considerations

Ethical issues (Including plagiarism, informed consent, misconduct, data fabrication and/or falsification, double publication and/or submission, redundancy, etc.) have been completely observed by the authors.
